# SENP6 induces microglial polarization and neuroinflammation through de-SUMOylation of Annexin-A1 after cerebral ischaemia–reperfusion injury

**DOI:** 10.1186/s13578-022-00850-2

**Published:** 2022-07-22

**Authors:** Meng Mao, Qian Xia, Gao-Feng Zhan, Qin-Jun Chu, Xing Li, Hong-Kai Lian

**Affiliations:** 1 Department of Anesthesiology and Perioperative Medicine, Zhengzhou Central Hospital Affiliated to Zhengzhou University, Zhengzhou, 450007 China; 2 Department of Anesthesiology, Tongji Hospital, Tongji Medical College, Huazhong University of Science and Technology, Wuhan, 430030 China; 3 Trauma Research Center, Zhengzhou Central Hospital Affiliated to Zhengzhou University, Zhengzhou, 450007 China; 4 Center for Advanced Medicine, College of Medicine, Zhengzhou University, Zhengzhou, 450007 China

**Keywords:** SENP6, Annexin-A1, SUMOylation, Microglial polarization, Neuronal damage, Cerebral ischaemia–reperfusion injury

## Abstract

**Background:**

Previous data have reported that Sentrin/SUMO-specific protease 6 (SENP6) is involved in ischaemic brain injury and induces neuronal apoptosis after cerebral ischaemia, but the role of SENP6 in microglia-induced neuroinflammation and its underlying mechanism remain poorly understood. This research systematically explored the function and potential mechanism of SENP6 in microglia-induced neuroinflammation after ischaemic stroke.

**Results:**

We first identified an increased protein level of SENP6 in microglia after cerebral ischaemia. Then, we demonstrated that SENP6 promoted detrimental microglial phenotype polarization. Specifically, SENP6-mediated de-SUMOylation of ANXA1 targeted the IκB kinase (IKK) complex and selectively inhibited the autophagic degradation of IKKα in an NBR1-dependent manner, activating the NF-κB pathway and enhancing proinflammatory cytokine expression. In addition, downregulation of SENP6 in microglia effectively reduced cocultured neuronal damage induced by ischaemic stroke. More importantly, we employed an AAV-based technique to specifically knockdown SENP6 in microglia/macrophages, and in vivo experiments showed that SENP6 inhibition in microglia/macrophages notably lessened brain ischaemic infarct size, decreased neurological deficit scores, and ameliorated motor and cognitive function in mice subjected to cerebral ischaemia surgery.

**Conclusion:**

We demonstrated a previously unidentified mechanism by which SENP6-mediated ANXA1 de-SUMOylation regulates microglial polarization and our results strongly indicated that in microglia, inhibition of SENP6 may be a crucial beneficial therapeutic strategy for ischaemic stroke.

**Graphical Abstract:**

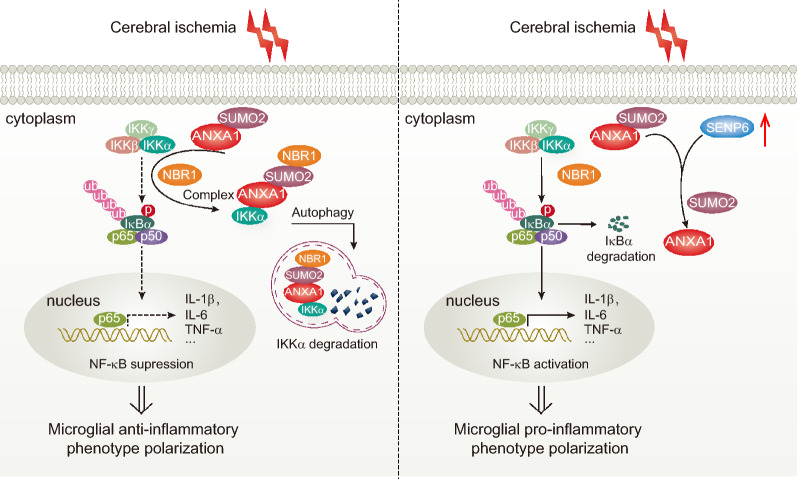

**Supplementary Information:**

The online version contains supplementary material available at 10.1186/s13578-022-00850-2.

## Introduction

Cerebral ischaemia, which accounts for 80–85% of all strokes, has long been recognized as the predominant cause of mortality and disability worldwide [1, 2]. In recent decades, the limited effective treatments for acute ischaemia have been intravenous thrombolysis and thrombectomy, which aim to restore blood flow during a limited treatment window [3, 4]. Nevertheless, there are no other safe and effective strategies for patients once the optimal therapeutic time window is missed; therefore, it is urgently needed to search for new therapeutic treatments. In recent decades, a substantial number of studies have identified that the postischaemic inflammatory response contributes significantly to the pathophysiological development of cerebral ischaemia [5, 6]. Thus, elucidating the precise mechanism of these inflammatory responses would reveal potential targets for the treatment of cerebral ischaemia.

Considerable evidence has shown that microglia, the main resident immune cells of the central nervous system (CNS), play essential roles in CNS development, maintenance and repair after many types of brain damage, including ischaemic stroke [7]. They act as guardians regulating immune and inflammatory responses and are activated under conditions of injury or infection. Activated microglia can be divided into two phenotypes, defined as the classically activated proinflammatory phenotype named M1 and the alternatively activated anti-inflammatory phenotype named M2 [8]. Classical M1-type microglia tend to express and release inflammatory cytokines, such as IL-1, iNOS, and TNF-α, which aggravate tissue damage after cerebral ischaemia. In contrast, M2-type microglia exhibit a neuroprotective effect by secreting anti-inflammatory cytokines, such as IL-4, IL-10, and TGF-β, which alleviate inflammatory responses and promote tissue repair [9]. On the basis of different phenotypic polarizations, activated microglia function as a ‘double-edged sword’ in ischaemic injury by displaying neurotoxic or neuroprotective effects [10, 11]. Therefore, identifying the potential mechanism of microglial polarization from a proinflammatory phenotype to an anti-inflammatory phenotype after cerebral ischaemia may be an effective therapeutic strategy for ischaemic stroke.

SENP6 is a member of the sentrin/SUMO-specific protease (SENP) family, including six members (SENP1–3, 5–7) that deconjugate SUMOs from substrates during the SUMOylation modification process [12, 13]. Numerous studies have shown that SENP6, by specifically depolymerizing SUMO chains, plays a pivotal role in regulating cellular protein function, expression, activity, localization, and stability [14, 15]. Studies have identified that SENP6 has a large effect on tumorigenesis, such as lymphomagenesis [16]. In addition, it was also proven that SENP6 played an important role in inflammatory responses. For example, SENP6-mediated de-SUMOylation of NF kappa-B essential modulator (NEMO) dampened NF-κB activation, further restraining neuroinflammation [17]. Additionally, our previous study found that SENP6 in neurons promoted neuronal apoptosis by mediating the de-SUMOylation of Annexin-A1 (ANXA1) after ischaemic stroke [18]. On the other hand, we also confirmed that the SUMOylation of ANXA1 could regulate microglial polarization after cerebral ischaemia. Briefly, SUMOylated ANXA1 acts as an adaptor to increase the interaction between IKKα and NBR1, thereby forming a complex to promote the selective autophagic degradation of IKKα and inhibiting NF-κB signalling pathway activation, eventually leading to anti-inflammatory phenotype polarization of microglia [19]. However, the role of SENP6 in microglial polarization induced by cerebral ischaemia has not been elucidated.

In this study, we attempted to investigate the role of SENP6 in microglial polarization and explore its underlying mechanism. Cerebral ischaemia induced the upregulation of SENP6 and enhanced the de-SUMOylation of ANXA1 mediated by SENP6. Further experiments showed that SENP6 induced microglial polarization to a proinflammatory phenotype and aggravated neuronal damage by promoting NF-κB signalling pathway activation via the de-SUMOylation of ANXA1. Specific intervention of SENP6 activity in microglia could reduce neurological injury after cerebral ischaemia, suggesting that intervention with SENP6 may be a new and promising strategy for ischaemic stroke treatment.

## Materials and methods

### Animals

C57BL/6J male mice (weighting 22–25 g, 8 weeks old) were purchased from Beijing River Laboratory Animal Corp. Ltd. The mice were raised in specialized boxes that were placed in a quiet room with a light–dark cycle of 12–12 h, a temperature controlled at approximately 22 °C, and free water and food. The animal experiments were approved by the Ethics Committee for Animal Experimentation of Zhengzhou Central Hospital Affiliated to Zhengzhou University (Zhenzhou, China) in accordance with the Animal Research: In vivo Reporting of Experiments (ARRIVE) guidelines. The data were analysed to detect significant differences with PASS (power analysis & sample size) software using a significance level of α = 0.05 with 80% power. All experiments were randomized, and the operator was blinded to the data analysis and experiment.

### Reagents and antibodies

Primary antibody: anti-β-actin (sc-47778, 1:1000), anti-HA (sc-7392, 1:1000) and anti-GFAP (sc-33673, 1:200) were obtained from Santa Cruz Biotechnology (Dallas, TX, USA); Anti-iNOS (18,985–1-AP, 1:500) was purchased from Proteintech Group (Wuhan, China); anti-CD16/32 (AF1460, 1:500) was purchased from R&D systems (Minneapolis, MN, USA); anti-NF-κB p65 (#8242, 1:1000), anti-IKKα (#11,930, 1:1000), anti-Phospho-NF-κB p65 (#3033, 1:1000), anti-Phospho-IKKα/β (#2697, 1:1000), anti-Phospho-IκBα (#2859, 1:1000), anti-IκBα (#4814, 1:1000), anti-NBR1(#9891, 1:1000), anti-cleaved PARP (5625, 1:1000), anti-cleaved caspase-3 (9664, 1:1000) and anti-cleaved caspase-9 (#20,750, 1:1000) were obtained from Cell Signaling Technology (Danvers, MA, USA); anti-SENP6 (HPA024376, 1:1000) were purchased from Sigma–Aldrich (St. Louis, MO, USA); anti-Iba1 (ab283319, 1:100) was obtained from Abcam (Boston, MA, USA) and anti-NeuN (MAB377, 1:200) was purchased from Millipore Biotechnology (Schwalbach, Germany). Protein A + G agarose beads were obtained from Beyotime Biotechnology (Shanghai, China), and Ni^2+^-NTA agarose was purchased from QIAGEN (Dusseldorf, Germany). CHX was obtained from Calbiochem (508,739; Darmstadt, Germany).

### Transient focal cerebral ischaemia

The intraluminal filament technique was used to induce focal cerebral ischaemia through the transient occlusion of the middle cerebral artery as we reported previously [20]. In brief, mice were anaesthetized using chloral hydrate (350 mg/kg, i.p.). To maintain the rectal temperature at approximately 37 ± 0.5 °C, the mice were placed on a thermostatic blanket (Harvard instrument, Holliston, MA, USA). The left common carotid artery (CCA), external carotid artery (ECA) and internal carotid artery (ICA) were exposed. Ligation of the CCA was performed with surgical nylon monofilament near the distal end of the CCA and ligation of the ECA was conducted at two positions at the end of ECA and near ICA and ECA bifurcations. Then, using ophthalmic scissors, a small incision was made between the nylon filament (0.22 to 0.23 mm in diameter), and the two ECA ligatures of double diameter lengths were gently inserted into the ICA from the ECA stump and advanced to the anterior cerebral artery (ACA) until slight resistance was felt. Successful occlusion was verified by laser Doppler flowmetry (PeriFlux System 5000, PERIMED, Sweden). After 60 min, the filament was withdrawn softly. The sham mice underwent the same surgical operation, but no embolus was inserted.

### Cell culture, transfection and OGD/R procedure

Primary microglia were cultured according to our previously reported method [19]. Briefly, mixed glial cells were isolated from the whole brains of C57BL/6 neonatal mice at postnatal days P1 and P2 and then cultured in high-glucose DMEM (Gibco, Gaithersburg, MD, USA) with 1% penicillin–streptomycin and 20% foetal bovine serum (FBS; Gibco) in a 5% CO_2_ incubator at 37 °C for 7 days. After 7 days, fresh complete medium was used to replace the old medium every 3 days. After 10–14 days post-dissection, primary microglia were gathered from the mixed glial cultures by shaking at 400 rpm for 4 to 6 h at 37 °C in a rotary shaker and seeded into poly-d-lysine (Sigma–Aldrich)-coated six-well plates at a density of 1 × 10^6^ per well for 24 h attachment before further treatment.

HEK293T cells were purchased from the American Type Culture Collection and grown in DMEM supplemented with 1% penicillin–streptomycin and 10% FBS at 37 ℃ in a 5% CO_2_-containing atmosphere. Following the manufacturer’s instructions, approximately 80%-90% confluent cell layers were transfected with plasmids by using Lipofectamine 3000 (Invitrogen, NY, USA). OGD/R was carried out as we described before. Typically, primary microglial cells and HEK293T cells were taken from a CO_2_ incubator, and glucose-free DMEM (Gibco) preheated to 37 ℃ was used to replace the cell culture medium. Next, the cells were placed in an oxygen-deprived (94%N_2_/5%CO_2_/1%O_2_) incubator at 37 °C for 1 h to establish OGD conditions. Finally, glucose-free DMEM was discarded, and then high-glucose DMEM was added to the cell plates to reoxygenate the cells under normoxic conditions for 24 h for subsequent assays.

### Protein extraction and preparation, immunoprecipitation and immunoblot analysis

Protein extraction and subcellular separation were carried out using the method reported previously [21]. Whole-cell lysates were prepared in radioimmune-precipitation assay (RIPA) lysate (Beyotime Biotechnology) supplemented with cOmplete™ protease inhibitor cocktail tablets (5 mg/ml; Roche Diagnostics, Rotkreuz, Switzerland). The NE-PER Nuclear and Cytoplasmic Extraction Reagent Kit (Thermo Fisher Scientific, Waltham, MA, USA) was chosen to extract nuclear and cytoplasmic fractions according to the manufacturer’s instructions. Then, a BCA protein assay kit (Beyotime Biotechnology) was applied to determine the protein concentrations of the above extracts. For immunoprecipitation, cells were lysed in immunoprecipitation (IP) buffer supplemented with NEM (N-ethylmaleimide, Sigma–Aldrich) or a protease inhibitor mixture. Then, anti-HA, anti-His, anti-NBR1 and anti-IKKα antibodies were added to suitable cell lysates, followed by incubation for 12 h using a shaker at 4 °C. After that, cell lysates with antibodies were incubated for 4 h at 4 °C with protein A/G plus agarose (Santa Cruz) and washed three times with precooled PBS buffer. Then, 2 × SDS–PAGE loading buffer was added to the samples followed by boiling at 95 °C for 5 min, and immunoblot analysis was performed to detect the protein expression. For immunoblotting, the protein samples were separated using SDS–PAGE. Then, the proteins were transferred to a polyvinylidene difluoride membrane (Roche Diagnostics) and blocked with 5% bovine serum albumin (BSA) for 60 min at room temperature. Finally, the membranes were probed using primary antibodies overnight at 4 °C. Horseradish peroxidase–conjugated secondary antibodies (1:20,000) were used. A chemiluminescence substrate kit (Thermo Pierce, Rockford, IL, USA) was used to perform immunodetection.

### Ni^2+^-NTA affinity purification

As reported previously, SUMOylated ANXA1 under the condition of denaturation was affinity-purified by Ni^2+^-NTA pull down [19]. In brief, plasmids expressing HA-tagged ANXA1, Myc-tagged SENP6 and His-tagged SUMO2 were transfected into HEK293T cells. After 24 h, the cells were taken from the incubator, washed twice with cold PBS buffer, and then treated with 800 mL Ni^2+^-NTA denatured buffer (10 mM Tris; 20 mM NEM; 6 M Gu-HCl and 100 mM NaH_2_PO_4_, pH 8.0). The samples were treated with ultrasound (2 × 20 s) to cut the DNA and then cleared via centrifugation (15,000×*g*, 10 min, 4 °C). The supernatant was mixed with 50 mL prewashed Ni^2+^-NTA agarose (Qiagen, Dusseldorf, Germany) and rotated at 4 °C to incubate for 3 h. Finally, 1 mL Ni^2+^-NTA washing buffer (0.1% Triton X-100; 100 mM NaH_2_PO_4_; 10 mM Tris/HCl, pH 6.3; 8 M urea) was added to wash the beads, and lastly eluted by boiling the beads in 50 μL 2 × SDS–PAGE loading buffer mixed with 200 mM imidazole for 5 min.

### LDH release and cell survival assays

According to the manufacturer’s instructions, an LDH Cytotoxicity Assay Kit (Beyotime Biotechnology) was employed to determine LDH release from primary microglial cells. For the survival assays, cells were fixed with 4% formaldehyde for 30 min and then permeabilized with 0.1% Triton X-100 for 15 min. Next, the primary neurons were incubated using a 50 μL /well /24-well plate TUNEL reaction mixture at 37 °C for 1 h and with 50 μl /well /24-well plate 1 × DAPI for 10 min at room temperature. Finally, the cells were examined, and TUNEL-positive cells were counted using fluorescence microscopy (IX73, Olympus, Tokyo, Japan).

### RNA extraction, reverse transcription, and quantitative real-time PCR (RT–qPCR)

Total RNA was extracted from primary microglial cells with TRIzol reagent (Invitrogen) according to the manufacturer’s instructions, and the RNA concentration was measured using spectrophotometry (Thermo Fisher Scientific). Next, the reverse transcription of the complementary DNA (cDNA) from 1 μg total RNA was performed by the ReverTra Ace-α-First Strand cDNA Synthesis Kit (Toyobo, Osaka, Japan). According to the manufacturer’s instructions, quantitative real-time PCR was conducted using a StepOnePlus Real-Time PCR System (Applied Biosystems, Foster City, CA, USA) and SYBR Green PCR Master Mix (Applied Biosystems). The relative gene expression was normalized to β*-actin* mRNA levels, and the gene expression was evaluated using the 2^−ΔΔCt^ method. The primer sequences are listed in Additional file 5: Table S1.

### Luciferase reporter assay

HEK293T cells plated in 96-well plates were transfected with NF-κB luciferase reporter and plasmids carrying vector, SENP6, sh. NC or sh. SENP6 with pRL-Renilla as a control. The cell lysate was collected after transfection for 48 h, and the Dual-Luciferase kit (Promega, Madison, WI, USA) was used according to the manufacturer’s instructions to detect the luciferase activity. Then, firefly luciferase activity was normalized to Renilla luciferase using the internal transfection efficiency control. The experiments were conducted in triplicate.

### Plasmid construction

Plasmids were constructed as we previously described [20]. PCR technology was used to amplify the full-length DNA segment ANXA1 and SUMO2 coding sequences, which were cloned into HA-tagged pcDNA 3.0 (HA-ANXA1) and Myc-pcDNA 3.0 (Myc-SUMO2), respectively. Similarly, His-tagged SENP6 was constructed by cloning the full-length DNA segment coding sequence into His-pcDNA 3.0 (His-SENP6). Wild-type SENP6 and shRNAs against SENP6 plasmids were purchased from Sangon Biotechnology Company (Shanghai, China). The plasmids were all transfected into HEK293T cells with a Lipofectamine 3000 reagent kit according to the manufacturer’s instructions.

### Viral vector production and transduction

Adenoviruses encompassing vector, wild-type SENP6, scramble control and shRNAs against SENP6 purchased from Vigene Biosciences (Jinan, China) were employed to infect primary cultured microglia. The sequences of shRNA were designed and verified as follows: mSENP6, 5′-GGG CAA ATC TAC TCA GTG TAG-3′. Primary cells were infected using diluted recombinant adenovirus at an optimal multiplicity of infection of approximately 50:1 to 100:1 according to our preliminary tests. After 48 h of transfection, the cells were subjected to subsequent experiments.

The adeno-associated virus expressing SENP6 shRNA (AAV2/6-CMV-sh. SENP6) and control adeno-associated virus (AAV2/6-CMV-sh. NC) were purchased from Genechem (Shanghai, China). As we performed previously, mice were anaesthetized with an intraperitoneal injection of 5% 350 mg/kg chloral hydrate and then fixed on a stereotaxic apparatus. A drill was used to perforate burr holes into the skull, and a stepper-motorized microsyringe (Hamilton, Reno, NV, USA) was used to inject 500 nl of virus solution into the injection sites in the area of the hippocampal CA1 region, cerebral cortex and striatum of the left hemisphere. After injection, the needle was kept in place for 5 min and then removed slowly. Four weeks later, mice that underwent virus injection were subjected to MCAO/R surgery for subsequent experiments. Injection into the hippocampus CA1, cerebral cortex and striatum were, respectively at coordinates anteroposterior (AP) − 2.00 mm, lateral (L) − 1.55 mm and dorsoventral (DV) − 1.55 mm; anteroposterior (AP) 0.00 mm, lateral (L) − 2.05 mm and dorsoventral (DV) − 1.50 mm; anteroposterior (AP) 0.14 mm, lateral (L) − 2.28 mm and dorsoventral (DV) − 3.50 mm.

### Neurological score

The neurological score was determined as we have previously described [22]. After 24 h of MCAO and reperfusion, the modified neurological severity score (mNSS) was selected to assess neurological dysfunction, which includes beam balance tests (scored 0 to 6), reflexes absent & abnormal movements (scored 0 to 2) and motor tests (including flexion of forelimb, flexion of hindlimb and head movement, scored 0 to 6). Accumulative scores of 1 to 4, 5 to 9, and 10 to 14 indicated slight, moderate, and serious damage, respectively. Neurological performance was evaluated by independent blinded researchers.

### TTC (2,3,5-triphenyltetrazolium chloride) staining

Approximately 24 h after MCAO surgery, the mice were euthanized. Their brains were extracted carefully and then frozen for 5 min at − 20 °C. The whole brain was cut into 6 consecutive approximately 2-mm-thick coronal slices from the frontal tips using a mouse brain matrix. Then, the cells were dyed using 2% TTC (Sigma–Aldrich). The brain slices were fixed using 4% paraformaldehyde after incubating for 20 min at 37 °C. ImageJ analysis software (NIH, Baltimore, MD, USA) was selected to measure the infarct area, which was the white region, and the normal tissue, which was the dark red region. The infarct size (%) was calculated using the equation: infarct size (%) = (contralateral area − ipsilateral noninfarct area)/contralateral area × 100%.

### Adhesive-removal somatosensory test

Mice were subjected to the adhesive removal test to evaluate somatosensory and motor deficits. In the process of the experiment, sensory deficits were detected in the latency for animals to become aware of the sticker on their paws, while motor deficits were detected in the latency for animals to remove the stickers. Briefly, animals in the experiment were placed in an empty cage and given 1 min to adjust to the tests. Adhesive tape was cut into small squares and then pasted on the animals’ paws. Next, the time of the mouse to first notice the stickers or remove the stickers was observed and recorded. A time limit of 120 s was enforced. The experiments were repeated continuously four times with a 10-min break after each trial, and the data were statistically analysed.

### Morris Water Maze (MWM) test

During this study, we conducted MWM tests to measure spatial learning and memory as reported previously [23]. Briefly, the water maze contained a circular pool 60 cm high and 120 cm in diameter and a round platform 6 cm in diameter and was filled with water submerging the platform 1 cm at 22 ± 2 °C. Different shapes as spatial reference clues were placed around the tank. Above the maze, a digital tracking device was used to record mouse swimming traces. Before the tests started, the mice were habituated to the testing room for 24 h. In the first 6 consecutive days, each group performed four sessions in turn to search for the submerged platform within 60 s. In the condition of a mouse not finding the platform in the allowed time, we guided it to the platform and allowed it to stay there for 10 s. Finally, the platform was removed after completing the training on the seventh day, and the animal traces in 60 s were recorded by a digital tracking device (Xinruan Information Technology, Shanghai, China), which automatically analysed the time to cross the platform area, the time the mice spent in the target quadrant and the latency time to reach the platform.

### The rotarod test

To evaluate motor function, a rotarod containing a 6-cm-diameter rotating cylinder with a coarse surface was used. Beforehand, the mice were placed in the testing environment for 30 min to acclimate. Then, the mice were placed in the centre and subjected to a training session at a speed of 5 to 10 rpm on the rotarod. Next, the speed of the rotarod was increased to 40 rpm within 5 min, during which time the mice fell off the rotarod. The experiments were repeated continuously four times with a break of 30 min after each trial, and the data were analysed statistically.

### Statistical analysis

Data from at least three repeated experiments are shown as the mean ± S.E.M. and the corresponding analysis was conducted using GraphPad Prism software (version 8.0.1, GraphPad Software, Inc.). The comparisons for more than two groups were performed using one-way or two-way analysis of variance (ANOVA) and repeated-measures ANOVA tests, which are shown in the figure legends. In addition, as indicated, Dunnett’s or Tukey’s post-hoc multiple comparison measurements were used for significant effects in variance analysis. The number of mice crossing over the platform location on Day 7 in the MWM test was analysed using the Kruskal–Wallis nonparametric test, followed by Dunnett’s post-hoc test. A *P* value lower than 0.05 was considered statistically significant.

## Results

### Microglial SENP6 expression was enhanced after cerebral ischaemia

**Fig. 1:**
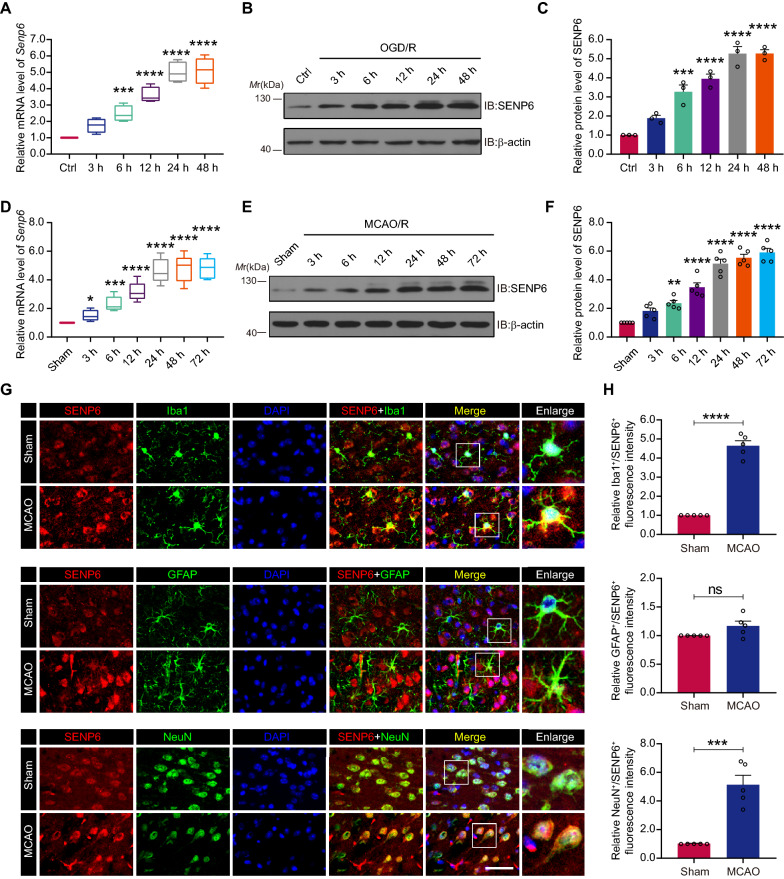
SENP6 is upregulated in microglia after cerebral ischaemia–reperfusion injury. **A** Primary microglia were challenged with OGD for 1 h and then reperfusion for 3 h, 6 h, 12 h, 24 h and 48 h. RT-qPCR confirmed the mRNA levels of *Senp6*. **B** The protein levels of SENP6 in primary microglia subjected to OGD treatment and reperfusion at the indicated times. **C** Quantitative analysis of the protein levels of SENP6 in **B**. **D** Density gradient centrifugation collected microglia around the ischaemic penumbra from mice for 3 h, 6 h, 12 h, 24 h and 72 h after MCAO surgery. RT–qPCR detected mRNA levels of *Senp6*. **E** The protein expression analysis of collected microglia by western blot assay. **F** Quantification of the protein levels of SENP6 in **E**. **G** Representative double immunostaining of SENP6 (red) with Iba-1 (a microglial marker, green), GFAP (an astrocyte glial marker, green) and NeuN (a neuronal marker, green) from ischaemic penumbra of brain tissue after MCAO surgery. **H** Quantification of Iba-1^+^/SENP6^+^, NeuN^+^/SENP6^+^, and GFAP^+^/SENP6^+^ fluorescence intensity was quantified using ImageJ. Scale bars, 40 µm. Data are presented as the mean ± S.E.M. from at least three dependent experiments and analysed by one-way ANOVA followed by Dunnett’s post-hoc test (**A**, **C**, **D**, **F**) or unpaired Student's t test (**H**). ns for *P* > 0.05, *P < 0.05, **P < 0.01, ***P < 0.001, and ****P < 0.0001

To clarify the role of SENP6 in microglia after cerebral ischaemia, we first detected the mRNA levels of SENP6 after OGD treatment in primary microglial cells and reoxygenation at 3 h, 6 h, 12 h, 24 h and 48 h. The RT–qPCR results revealed that the mRNA levels of *Senp6* increased at 3 h and peaked at 24 h after OGD treatment (Fig. 1A). Consistent with the mRNA results, immunoblot assays also indicated gradually increasing protein levels of SENP6, and the protein level peaked at 24 h after OGD treatment (Fig. 1B, C). To further explore the influence of ischaemia on SENP6 expression, we next measured SENP6 mRNA and protein levels in the brains of mice subjected to MCAO/R surgery. In accordance with the in vitro observations, RT–qPCR analysis and immunoblot assays also demonstrated that the mRNA and protein levels of SENP6 were progressively increased with longer periods of reperfusion (Fig. 1D-F). Furthermore, immunofluorescence staining of brain sections showed that SENP6 were primarily expressed in microglia and neurons and to a lesser extent in astrocytes (Fig. 1G). Moreover, these results were corroborated the RT-qPCR and immunoblots results, showing that SENP6 staining in microglia and neurons was more intense in the mice subjected to MCAO/R surgery (Fig. 1H). These results were also consistent with the data showed in our previous study that cerebral ischaemia induces the expression of SENP6 in neurons [18]. Taken together, these data demonstrated that microglial SENP6 expression was upregulated after brain ischaemia.

### Inhibition of SENP6 induces microglial polarization to an anti-inflammatory phenotype after OGD/R treatment

**Fig. 2:**
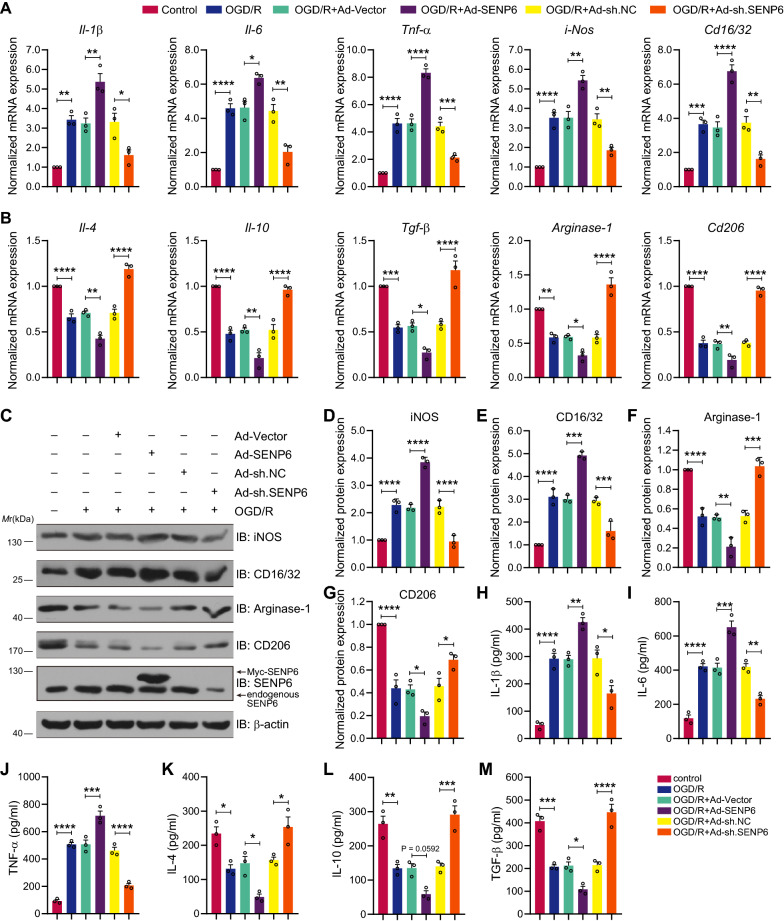
Inhibition of SENP6 promoted microglial polarization towards an anti-inflammatory phenotype after OGD/R treatment. **A**, **B** Primary cultured microglia were infected with adenovirus expressing wild-type SENP6 or shRNA against SENP6 following OGD/R treatment. The mRNA levels of OGD/R-induced proinflammatory (**A**) and anti-inflammatory (**B**) phenotype marker genes were detected by RT–qPCR. **C** Immunoblots showing the protein expression of iNOS, CD16/32, Arginase-1 and CD206 in primary microglia infected with Ad-SENP6 or Ad-sh. SENP6. **D**–**G** Quantification analysis of the protein levels in **C**. **H**–**M** The supernatant of primary microglia was collected, and cytokine levels were detected by ELISA. Data are presented as the mean ± S.E.M. from at least three dependent experiments and analysed by one-way ANOVA followed by Tukey’s post-hoc test. ns for *P* > 0.05, *P < 0.05, **P < 0.01, ***P < 0.001, and ****P < 0.0001

We then sought to explore the role of SENP6 in the CNS, especially its biological function in cerebral ischaemia-induced microglial polarization and neuroinflammation. Recombinant adenoviruses encoding vector (Ad-vector), wild-type SENP6 (Ad-SENP6), scramble control (Ad-sh. NC) or shRNA against SENP6 (Ad-sh. SENP6) were used to infect primary microglial cells to upregulate or downregulate SENP6 levels. The knockdown efficiency of Ad-sh. SENP6 is shown in Additional file 1: Figure S1. Then, we detected the functional mechanism of the effect of SENP6 on microglial polarization. First, we used an RT–qPCR assay to quantify proinflammatory marker genes, including *Il-1β*, *Il-6*, *Tnf-α*, *iNos*, and *Cd16/32*, and anti-inflammatory marker genes, including *Il-4*, *Il-10*, *Tgf-β*, *Arginase-1* and *Cd206*. The results demonstrated that proinflammatory genes in the OGD/R treatment group were significantly increased, while anti-inflammatory genes were markedly suppressed compared with the control group. In addition, overexpressing SENP6 further promoted OGD/R-induced proinflammatory gene expression and inhibited anti-inflammatory gene expression, while microglia infected with Ad-sh. SENP6 showed the opposite effects (Fig. 2A, B). These results were identified again by immunoblots that showed the protein expression levels of proinflammatory mediators (iNOS and CD16/32) and anti-inflammatory mediators (Arginase-1 and CD206) (Fig. 2C–G). To further confirm the role of SENP6 in microglial polarization, ELISA was performed to reveal the secretion of proinflammatory marker cytokines (IL-1β, IL-6, and TNF-α) and anti-inflammatory marker cytokines (IL-4, IL-10, and TGF-β), and the results were consistent with the mRNA expression results (Fig. 2H–M). Collectively, these results indicated that SENP6 plays a significant role in regulating the OGD/R-induced phenotypic polarization of microglial cells.

We next aim to explore the contribution of de-SUMOylated ANXA1 in the detrimental roles of SENP6 in microglia-related neuroinflammation after ischaemic brain injury. To block the de-SUMOylation of ANXA1 mediated by SENP6, we used a SUMO2 fusion-directed SUMO modification system to allow for the efficient and selective in vivo SUMOylation of ANXA1 for further investigation. To this end, SUMO2 was fused to the C terminus of wild-type ANXA1 (a construct named ANXA1-SUMO2) as we previously reported [18, 19]. Primary cultured microglia were infected with recombinant adenoviruses encoding SENP6 or together with ANXA1-SUMO2, and then subjected to OGD/R. The ELISA assay results showed that overexpressing SENP6 enhanced OGD/R-induced pro-inflammatory marker cytokines secretion, but inhibited anti-inflammatory marker cytokines secretion. However, these effects were significantly reversed by ANXA1-SUMO2 overexpression (Additional file 2: Figure S2). These data indicated that SENP6-dependent neuroinflammation after ischaemic brain injury may depend on its enzymatic activity to mediate the de-SUMOylation of ANXA1.

### SENP6 promotes the activation of the NF-κB signalling pathway in microglial cells after OGD/R

**Fig. 3:**
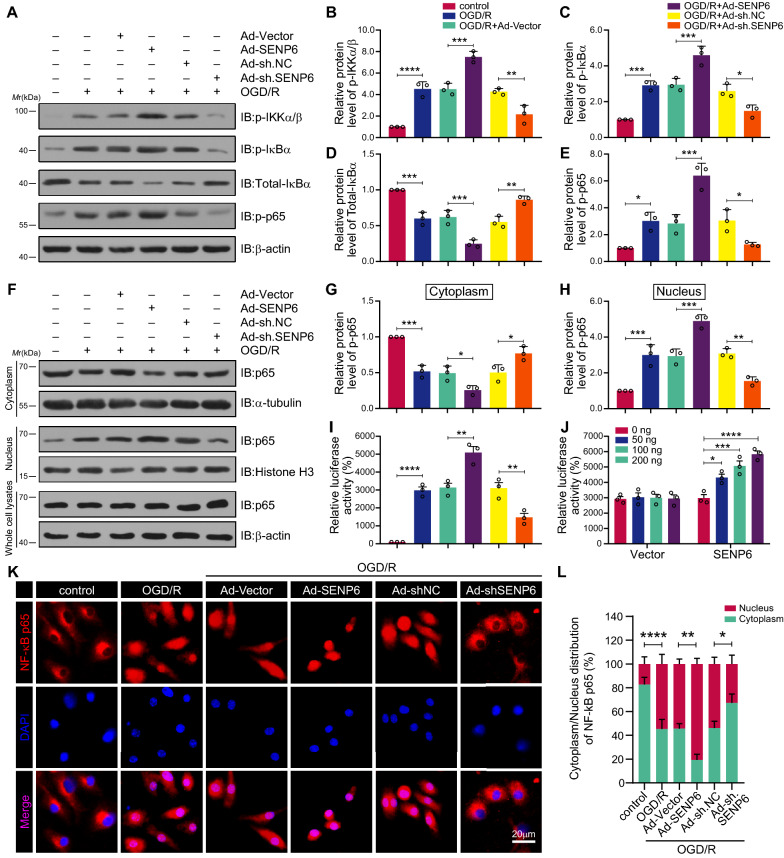
SENP6 downregulation decreased NF-κB signalling pathway activity in primary cultured microglia subjected to OGD/R. **A** Representative immunoblots showing the phosphorylation of IKKα/β, IκBα and p65 in microglia infected with adenovirus encoding vector, SENP6, sh. NC and sh. SENP6 following OGD/R or not. **B**–**E** Quantification of the p-IKKα/β, p-IκBα and p-p65 proteins in **A**. **F** Immunoblots detected NF-κB p65 in cytoplasmic and nuclear fractions of primary microglia, as well as in whole-cell lysates. **G**, **H** Quantitative analysis of NF-κB p65 protein levels in the cytoplasm and nuclear extracts of microglia in **F**. **I** The p65 transcriptional activity was confirmed by a Dual-Luciferase reporter assay in HEK293T cells transfected with a plasmid expressing SENP6 or sh. SENP6. **J** The change in p65 transcriptional activity in HEK293T cells with increasing SENP6 doses. **K** Immunofluorescence staining determined the subcellular localization of NF-κB p65 in primary microglia. **L** Statistical analysis of the fluorescence intensity in **K**. Data are presented as the mean ± S.E.M. from at least three dependent experiments. Data in **J**, **L** were analysed by two-way ANOVA, and all others were analysed using one-way ANOVA followed by Tukey’s post-hoc test. ns for *P* > 0.05, *P < 0.05, **P < 0.01, ***P < 0.001, and ****P < 0.0001

Our previous studies demonstrated that SUMOylated ANXA1 promoted microglial polarization towards an anti-inflammatory phenotype by suppressing NF-κB pathway activation after ischaemic stroke, and moreover, in a recent study, we provided experimental evidence that SENP6 could mediate ANXA1 de-SUMOylation [18, 19]. Therefore, we hypothesized that in microglial cells, SENP6 could promote de-SUMOylation of ANXA1, which would activate the NF-κB signalling pathway and eventually lead to microglial polarization towards a proinflammatory phenotype. To this end, we next explored the role of SENP6 in NF-κB signalling pathway activation. Here, we first employed immunoblots to detect the protein level of total IκBα and the phosphorylation of IKKα/β, IκBα and p65. The results suggested increased phosphorylation levels of IKKα/β, IκBα and p65 and reduced total IκBα protein expression after OGD/R stimulation. In addition, SENP6 overexpression notably enhanced the phosphorylation of IKKα/β, IκBα and p65 and the subsequent degradation of IκBα compared with the vector group under OGD/R conditions, while SENP6 knockdown showed the opposite effects (Fig. 3A–E). These results suggest that SENP6 promotes NF-κB signalling pathway activation.

Next, we further explored the nuclear translocation of p65. The immunoblot results showed that SENP6 overexpression improved the accumulation of p65 in the nucleus and reduced it in the cytoplasm after OGD/R. Conversely, knockdown of SENP6 significantly suppressed the nuclear translocation of p65 and led to its accumulation in the cytoplasm (Fig. 3F–H). Then, we measured the transcriptional activity of p65 via the Dual-Luciferase reporter assay. The results indicated that OGD/R treatment upregulated p65 transcriptional activity. Meanwhile, SENP6 overexpression enhanced the OGD/R-induced transcriptional activity of p65, and there was a dose-dependent activation effect of SENP6 on the transcriptional activity of p65. However, endogenous downregulation of SENP6 weakened its transcriptional activity under OGD/R stimulation (Fig. 3I, J). Finally, we conducted an immunofluorescence assay to determine the location of p65 in primary microglial cells. The results also confirmed that SENP6 promoted p65 nuclear translocation (Fig. 3K, L). Together, these data suggested that SENP6 in microglia aggravated the activation of the NF-κB pathway after cerebral ischaemia.

### SENP6-mediated de-SUMOylation of ANXA1 inhibited IKKα protein degradation

**Fig. 4:**
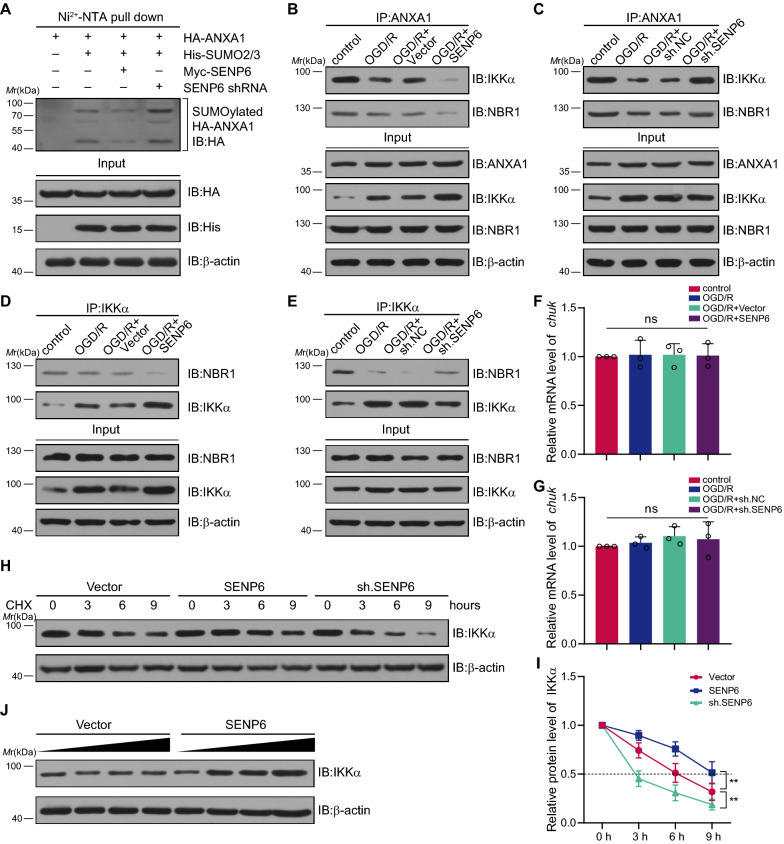
SENP6-mediated ANXA1 de-SUMOylation inhibits IKKα protein degradation. **A** Ni^2+^-NTA agarose affinity pull-down assay results representing ANXA1 SUMOylation in HEK293T cells transfected with plasmids expressing SENP6 or sh. SENP6. **B** Coimmunoprecipitation (co-IP) assay showing the interaction of ANXA1 with IKKα and NBR1 in primary cultured microglia infected with adenovirus encoding vector or SENP6. **C** Co-IP assay showing the interaction of ANXA1 with IKKα and NBR1 in primary cultured microglia infected with adenovirus encoding sh. NC or sh. SENP6. **D** Primary cultured microglia were infected with adenovirus expressing SENP6 or vector. The interaction between IKKα and NBR1 in microglia was evaluated by a co-IP assay. **E** A Co-IP assay was performed to explore the interaction of IKKα and NBR1 in microglia infected with sh. NC or sh. SENP6. **F** RT–qPCR assay results show the *Chuk* mRNA levels in microglia infected with adenovirus encoding vector or SENP6. **G** RT–qPCR shows the *Chuk* mRNA levels in microglia infected with adenovirus encoding sh. NC or sh. SENP6. **H** Sh. SENP6 cells reduce the half-life of ectopically expressed IKKα in primary microglia. **I** Quantitative analysis of the half-life of ectopically expressed IKKα in **I**. **J** Effects of increasing the dose of SENP6 on ANXA1-mediated degradation of endogenous IKKα. Data are presented as the mean ± S.E.M. from at least three dependent experiments and analysed by one-way ANOVA followed by Tukey’s post-hoc test (**F** and **G**) or repeated-measures (RM) ANOVA followed by Tukey’s post-hoc test (**I**). ns for *P* > 0.05, **P < 0.01

In our previous study, we identified that SUMOylated ANXA1 could act as an adaptor to enhance the binding of IKKα with the autophagy receptor NBR1, thereby promoting the degradation of IKKα through selective autophagy and ultimately inhibiting the NF-κB signalling pathway [19]. Considering the above, we hypothesize that SENP6 activation of the NF-κB signalling pathway may be dependent on its effect on IKKα degradation. To verify this, we first transfected HA-tagged ANXA1 and His-tagged SUMO2, along with Myc-tagged SENP6 or SENP6 shRNA, into HEK293T cells. The knockdown efficiency of SENP6 shRNA is shown in Additional file 3: Figure S3. Then, the Ni^2+^-NTA (nickel–nitrilotriacetic acid) agarose precipitation results showed that SENP6 overexpression reduced the SUMOylation level of ANXA1 (Fig. 4A). Next, primary cultured microglia were transduced with recombinant adenovirus encoding SENP6 or shRNA against SENP6. Coimmunoprecipitation (Co-IP) assay results revealed that SENP6 obviously inhibited the interaction between ANXA1, IKKα and NBR1, while SENP6 shRNA enhanced this interaction (Fig. 4B, C). To further confirm this result, we examined the effect of SENP6 on the interaction between IKKα and NBR1. As expected, SENP6 reduced the interaction between IKKα and NBR1, and SENP6 shRNA showed the opposite effect (Fig. 4D, E). In addition, RT–qPCR results revealed that both the overexpression and downregulation of SENP6 did not change the mRNA level of *Chuk,* which encodes the IKKα protein (Fig. 4F, G). Next, a cycloheximide (CHX) “chase” assay was conducted to detect the time course of SENP6-induced IKKα protein degradation. The results showed that SENP6 shRNA quickened the turnover of IKKα (Fig. 4H, I). Furthermore, there is a dose-dependent relationship between SENP6 and IKKα. The protein level of IKKα increased gradually with increasing SENP6 overexpression (Fig. 4J). Collectively, these results indicated that SENP6 blocks IKKα protein degradation.

### SENP6 exacerbates OGD/R-induced neuronal damage

**Fig. 5:**
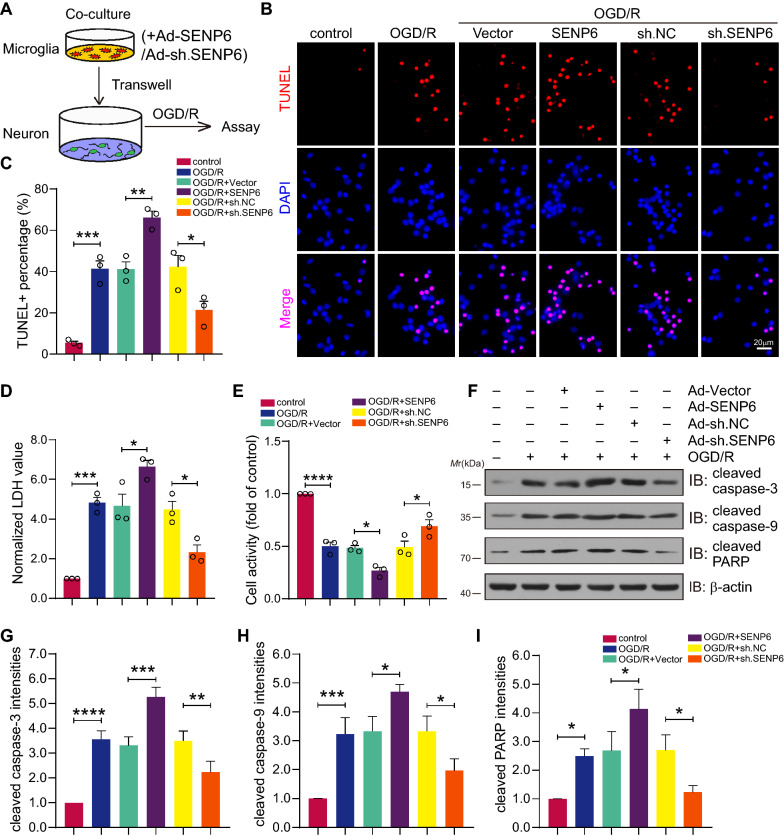
SENP6 overexpression in microglia promotes neuronal apoptosis under coculture conditions. **A** Primary cultured microglia were infected with adenovirus encoding the SENP6 coding sequence or shRNA sequence for 48 h and were cocultured with neurons, followed by OGD/R treatment. **B** Representative TUNEL staining labelling of apoptotic neurons. **C** Quantification of apoptotic neurons in **B**. **D** Collecting the supernatant in neuronal coculture. LDH assay indicating LDH release. **E** CCK-8 assay detecting neuronal cell viability when cocultured with adenovirus encoding SENP6 or sh. SENP6-infected primary microglia. **F** Immunoblots indicating the protein expression of representative apoptosis molecules in cocultured primary neurons. **G**–**I** Quantitative analysis of cleaved caspase-3, cleaved caspase-9, and cleaved PARP protein expression in **F**. Scale bar = 20 μm. Data are presented as the mean ± S.E.M. from at least three dependent experiments and analysed by one-way ANOVA followed by Tukey’s post-hoc test. *P < 0.05, **P < 0.01, ***P < 0.001, and ****P < 0.0001

We next examined the function of SENP6 inhibition in microglial hyperactivation-induced neuronal damage. Here, primary microglia and neurons were cocultured. Primary microglial cells were transduced with recombinant adenovirus carrying the SENP6 coding sequence or shRNA sequence (Fig. 5A). First, TUNEL staining showed that SENP6 upregulation in microglia aggravated neuronal apoptosis, and conversely, the apoptosis level of neurons was reduced when SENP6 was inhibited in cocultured primary microglia (Fig. 5B, C). The LDH release assay revealed that SENP6 overexpression increased LDH release, while SENP6 knockdown reversed it, which is consistent with the apoptosis results (Fig. 5D). Meanwhile, the cell-counting-kit-8 (CCK8) assay also indicated high cell viability of neurons after SENP6 inhibition in microglial cells (Fig. 5E). Finally, we detected the protein expression of several cell apoptotic markers, including cleaved caspase-3, cleaved caspase-9, and cleaved PARP. Immunoblots indicated that apoptotic marker protein expression was significantly increased in the Ad-SENP6 group under OGD/R treatment, while apoptotic proteins in the SENP6 knockdown group showed the opposite tendency (Fig. 5F–I). These results indicated that knockdown of SENP6 in primary macroglia obviously attenuated OGD/R-induced neuronal damage.

### SENP6 inhibition in microglia lessened infarct volume and ameliorated neurological functions after focal ischaemic injury

**Fig. 6:**
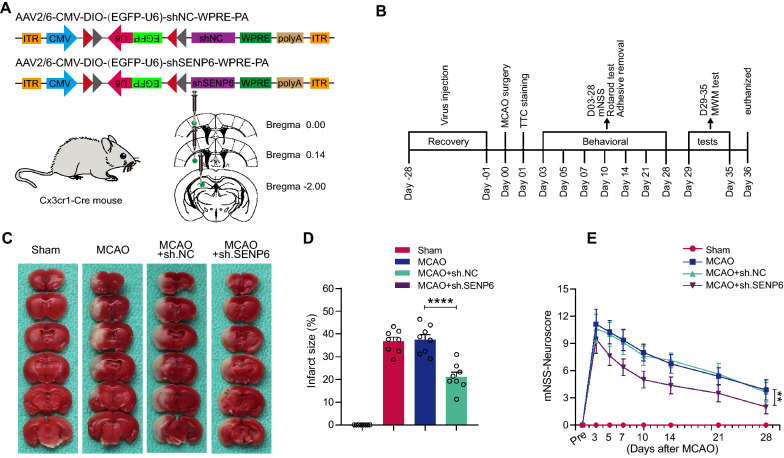
Knockdown of SENP6 in microglia/macrophages protects mice against focal ischaemic injury. **A** Schematic of adeno-associated virus vectors for SENP6 shRNA to downregulate SENP6 in microglia/macrophages. **B** Diagrammatic drawing of the whole experimental procedure. **C** Representative TTC staining indicating the ischaemia infarct volume. n = 8 mice in each group. **D** Quantification of infarct size in **C**. **E** Neurological deficit scores at different time points following MCAO surgery and reperfusion. Statistical differences in **E** were determined by RM ANOVA followed by Tukey’s post-hoc test. Data in **D** were assessed by one-way ANOVA followed by Dunnett’s post-hoc test. Data are presented as the mean ± S.E.M. n.s. for *P* > 0.05, *P < 0.05, **P < 0.01, ***P < 0.001 and ****P < 0.0001

In vitro, we demonstrated that inhibition of SENP6 could induce microglial polarization towards an anti-inflammatory phenotype, suppressing the secretion of inflammatory cytokines from microglia and alleviating the damage to cocultured neurons. Then, we investigated the protective effects of SENP6 knockdown on cerebral ischaemia in vivo. First, we applied an adeno-associated virus type 2/6 (AAV2/6)-mediated approach to knockdown SENP6 in microglia in the brains of ischaemic mice [19, 20, 24]. For this, we constructed AAV2/6, which expresses shRNA only in cells expressing Cre recombinase (Fig. 6A). Figure 6B displays the whole process of animal experiments. As indicated, AAV2/6 virus was injected into specific brain areas of Cx3cr1-Cre mice, including the hippocampal CA1 region, cerebral cortex, and striatum. Four weeks later, the mice that underwent MCAO surgery were employed to establish a transient focal ischaemia–reperfusion injury model. Then, according to the indicated time points, histological and behavioural studies were performed. We first examined whether AAV-mediated knockdown of SENP6 altered the phenotype polarization of microglia in ischaemic stroke mice. Microglia were isolated from ischaemic stroke animal model mice, and RT-qPCR was used to examine the mRNA level of phenotype marker genes. As shown in Additional file 4: Figure S4, the pro-inflammatory phenotype marker genes were markedly reduced, while the anti-inflammatory phenotype marker genes were significantly increased in SENP6 knockdown mice. These results indicated that SENP6 induce microglial pro-inflammatory polarization and neuroinflammation after MCAO in mice. We then detected and counted the infarct volume at 24 h after reperfusion via TTC staining. The results showed that the infarct volume of SENP6-knockdown mice was much smaller than that of negative control mice (Fig. 6C, D). Furthermore, we assessed neurological deficits using the modified neurological severity score (mNSS) and discovered that SENP6 knockdown effectively lowered the scores of mice (Fig. 6E).

### SENP6 inhibition in microglia improved neurobehavioral function after focal ischaemic injury

**Fig. 7:**
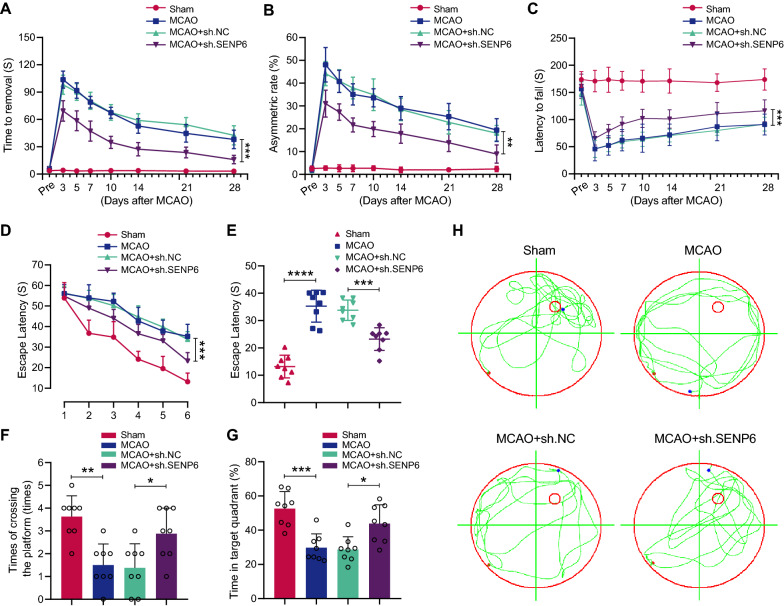
Knockdown of SENP6 in microglia/macrophages promotes cognitive and motor function following focal ischaemic injury in mice. **A** The time to removal in adhesive removal tests. **B** The asymmetric rate in cylinder tests. **C** The latency time to fall off the rotarod drum during the final test. **D**–**H**) Latency trial (**D**, **E**) and probe trial (**F**, **G**) results in the MWM tests. **D** Escape latency to reach the hidden platform during Days 1 to 6 of testing. **E** Exploration time spent on the hidden platform on Day 6. **F** The times of crossing over the target platform location in probe trials on Day 7. **G** The time spent in the target quadrant. **H** Representative swimming traces on Day 7. Statistical differences in **A**–**D** were determined by RM ANOVA followed by Tukey’s post-hoc test. Data in **F** were assessed by the Kruskal–Wallis nonparametric test, and all others were assessed by one-way ANOVA followed by Tukey’s post-hoc test. n = 8 mice per group. Data are presented as the mean ± S.E.M. n.s. for *P* > 0.05, *P < 0.05, **P < 0.01, ***P < 0.001 and ****P < 0.0001

We then performed the adhesive removal test and cylinder test to evaluate the sensorimotor functions of the experimental animals. The time that animals in the SENP6 inhibition group spent removing adhesive tape from their forepaws and their asymmetric rate in the cylinder test were evidently reduced (Fig. 7A, B). Moreover, we carried out rotarod tests to estimate animal motor function and found that inhibiting SENP6 in the brains of mice increased the time to fall (Fig. 7C). Finally, we detected the spatial learning and memory functions of mice after MCAO via the Morris water maze (MWM) test. The data revealed that mice that underwent MCAO surgery exhibited significant cognitive deficits, whereas SENP6-knockdown mice displayed significant cognitive improvement, including the latency to find the submerged platform, the time spent in the target quadrant, and the number of platform crossovers (Fig. 7D–G). Representative swimming tracings of the mice for the probe trials are presented in Fig. 7H. In contrast to the MCAO group, the search traces of the mice in the SENP6 knockdown group were more concentrated in the target quadrant or adjacent quadrants. Collectively, these results showed that SENP6 inhibition remarkably ameliorated MCAO-induced cognitive dysfunction.

## Discussion

Here, we investigated the role of SENP6 in microglial polarization after cerebral ischaemia and its underlying mechanism. First, we confirmed that cerebral ischaemia upregulated the level of SENP6 in microglia in vitro and ex vivo. Downregulating SENP6 in microglia could lead to microglial polarization from a proinflammatory phenotype induced by cerebral ischaemia to an anti-inflammatory phenotype. Importantly, our previous studies have identified that the SUMOylation of ANXA1 promotes microglial polarization and that SENP6 mediates the de-SUMOylation of ANXA1 [18, 19]. Based on these previous data, we then analysed the mechanism by which SENP6 affected microglial polarization and found that SENP6-mediated de-SUMOylation of ANXA1 blocked the degradation of IKKα, thereby promoting the activity of the NF-κB signalling pathway under ischaemia, eventually aggravating the apoptosis of neurons cocultured with microglia. In addition, this study also revealed that downregulation of SENP6 in microglia alleviated the neurological functional outcomes of mice subjected to cerebral ischaemic injury.

Accumulated data have demonstrated that SUMOylation is one of the diverse posttranscriptional modifications that regulates many protein functions [25, 26]. It has been verified that ANXA1 can be modified by SUMO2/3 and that this modification is pivotal for its protein functions [27, 28]. In our recent study, we also clarified that ANXA1 SUMOylation promoted microglial polarization to an anti-inflammatory phenotype under cerebral ischaemia [19]. SUMOylation modification is a dynamic process and can be reversed by SUMO proteases. The SUMO protease SENP6 is involved in deconjugating SUMO chains from substrate proteins and has specificity for SUMO2/3 [29, 30]. SENP6 is important in regulating genome stability, cell proliferation, and autoimmune responses [31, 32]. In a previous study, we identified that SENP6 aggravated neuronal apoptosis by de-SUMOylating ANXA1 after ischaemic stroke [18]. However, whether and how SENP6 contributes to microglial polarization remains unclear. In the present study, we discovered that SENP6 overexpression in microglia enhanced proinflammatory cytokine expression and aggravated neuronal damage. Some studies have also verified the proinflammatory role of SENP6. For example, Rachael et al*.* demonstrated that SENP6 and SENP7 mediate the de-SUMOylation of NLRP3 and activate inflammasomes, thereby increasing the inflammatory response [33]. In addition, a study also showed that SENP6 shortened the half-life of IκBα and induced inflammation by activating the NF-κB signalling pathway [34]. However, it was also reported that SENP6 could negatively regulate inflammation caused by lipopolysaccharide by boosting NF-κB activation in microglia and TLR4-induced NF-κB signalling by mediating the de-SUMOylation of NEMO [35, 36]. The different functions of the SENP6 response to inflammation might be related to different disease models or cell types.

Inflammation is significantly triggered after ischaemic stroke and throughout the whole progression of cerebral ischaemia–reperfusion, in which microglia function as the main immune cells that are activated to a proinflammatory phenotype, typically releasing proinflammatory cytokines and exacerbating nerve injury [37]. Therefore, promoting microglial polarization from the noxious proinflammatory type to the favourable anti-inflammatory type may be a prospective treatment method for stroke. Here, we further found that inhibition of SENP6 promoted microglial polarization to an anti-inflammatory phenotype. Indeed, inhibition of SENP6 in microglia increased the SUMOylation status of ANXA1, which facilitated the combination of IKKα with NBR1 to mediate IKKα degradation, thus inhibiting NF-κB signalling pathway activity and ultimately restraining proinflammatory cytokine expression. Moreover, the phagocytosis of microglia also plays a crucial role in the cerebral immune response after ischaemic stroke [38]. For instance, it was demonstrated that microglial phagocytosis mediated by the P2Y6 receptor could alleviate neurological damage induced by ischaemic stroke [39]. It was also reported that TREM2 enhanced the phagocytic activity of microglia and significantly attenuated ischaemic injury in experimental stroke [40]. However, whether SENP6 influences microglial phagocytosis after ischaemic stroke remains unclear and needs to be further explored.

Accumulated studies have identified that the NF-κB signalling pathway not only plays a crucial role in regulating the expression of many proinflammatory cytokines but is also the key signalling pathway for microglial polarization [41, 42]. Evidence from our research demonstrated that SENP6 deficiency obviously suppressed the activity of the NF-κB signalling pathway. The current study shows that the NF-κB signalling pathway can directly regulate the transcription of proinflammatory cytokines, including IL-1β, iNOS, and TNF-α, while inhibiting SENP6-mediated ANXA1 de-SUMOylation-regulated anti-inflammatory mediators may result from its effects on the NF-κB signalling pathway. The IKK complex is critical for NF-κB activation, and it contains three subunits (catalytic subunits IKKα and IKKβ, and regulatory subunit NEMO). Extensive studies have verified that the phosphorylated degradation of IκB mediated by IKKα/β plays a key role in NF-κB signalling pathway activation, subsequently resulting in NF-κB nuclear transport and related gene expression [43, 44]. Our results showed that SENP6-mediated ANXA1 de-SUMOylation specifically blocked the degradation of IKKα and then enhanced the activity of the NF-κB pathway and the subsequent expression of multiple proinflammatory cytokines. There is also a study that showed that SENP6 acting on IκBα increased NF-κB activation induced by radiation, which is in accordance with our results [34], but this study is the first to identify that SENP6 could influence NF-κB activation via IKKα.

In the present study, we further verified the protective neurological role of inhibiting SENP6 in microglia in a mouse focal cerebral ischaemia model by developing an AAV-based method to knockdown SENP6 in microglia/macrophage cells in the CA1 region, cortex, and striatum of the brain; nonetheless, as the insertion of AAV was performed before animals underwent focal cerebral ischaemia surgery for 4 weeks, postischaemic administration to evaluate the therapeutic effect requires further validation. Therefore, identifying SENP6-specific inhibitors or active substances that specifically intervene in the SENP6-ANXA1 interaction may be a prospective and effective therapeutic approach. On the other hand, we injected AAV particles into Cx3cr1-Cre mice expressing Cre recombinase under the direction of the Cx3cr1 promoter in the mononuclear phagocyte system, which contains monocytes, macrophages, and microglia [45, 46]. Thus, the AAV particles not only infect microglia but also infiltrating macrophages and monocytes, and the function of AAV particle injections on macrophages and monocytes remains unclear and needs to be explored in the future.

## Conclusions

In summary, our research uncovered a cellular mechanism by which SENP6 participates in microglial polarization. We have provided profound evidence showing that SENP6 mediates the de-SUMOylation of ANXA1, thereby inhibiting the autophagic degradation of IKKα in an NBR1-dependent manner, activating the NF-κB signalling pathway and inducing the microglial inflammatory response after cerebral ischaemia, while specifically inhibiting SENP6 decreased proinflammatory mediator expression and increased anti-inflammatory mediator expression, thereby protecting experimental animals against neurological damage. Overall, this study clarified a previously undiscovered role of SENP6 and indicated that SENP6 inhibition promoting microglial polarization to an anti-inflammatory phenotype may be a new and promising therapeutic method for ischaemic stroke or other neuroinflammatory diseases and disorders.

## Supplementary Information

**Additional file 1: Fig. S1.** The interference efficiency of Ad-sh. SENP6 against mouse SENP6. **A** Primary cultured microglia were transfected with adenoviral particles expressing either negative control (NC) or SENP6-targeting shRNA for 48 h. Western blotting was performed to examine the blocking efficiency. (**B**) Statistical analysis of the data shown in Figure S1A. The data are expressed as the means ± S.E.M. from three independent experiments. ***P < 0.001 versus sh.NC.

**Additional file 2: Fig. S2.** SENP6 induced neuroinflammation after ischaemic brain injury depending on its de-SUMOylation of ANXA1. Primary cultured microglia were infected with adenovirus expressing SENP6 or together with ANXA1-SUMO2 following OGD/R treatment. The cytokine levels of OGD/R-induced pro-inflammatory (A to C) and anti-inflammatory (D to F) phenotype marker genes were detected by ELISA assay. Data are presented as the mean ± S.E.M. from three dependent experiments and analysed by one-way ANOVA followed by Tukey’s post hoc test. *P < 0.05, **P < 0.01, ***P < 0.001, and ****P < 0.0001.

**Additional file 3: Fig. S3.** The interference efficiency of sh. SENP6 against human SENP6. **A** HEK293 cells were transfected with shRNA plasmids expressing negative control (NC) or SENP6-targeting shRNA. The knockdown of endogenous SENP6 expression was confirmed by western blot analysis. (**B**) Statistical analysis of the data shown in Figure S2A. The data are expressed as the means ± S.E.M. from three independent experiments. **P < 0.01 versus sh.NC.

**Additional file 4: Fig. S4.** Microglia SENP6 silencing promotes an anti-inflammatory phenotype of microglia after cerebral ischaemic injury. (A, B) AAV mediated SENP6 silencing attenuated MCAO-induced mRNA expression of pro-inflammatory marker genes (A) and promoted the mRNA expression of anti-inflammatory marker genes (B) in microglia cells isolated from ischaemic stroke mice. The mRNA levels of pro-inflammatory and anti-inflammatory mediators were detected by RT-qPCR. The results were analysed by one-way ANOVA followed by Tukey’s post hoc test. Data are presented as the mean ± S.E.M., *P < 0.05, **P < 0.01, ***P < 0.001 and ****P < 0.0001.

**Additional file 5: Table S1.** Primers used in this study.

## Data Availability

The authors declare that all data supporting the findings of this study are available within the paper and its Additional files.

## Abbreviations

AAV: Adeno-associated virus
ACA: Anterior cerebral artery
Ad: Adenoviruses
ANXA1: Annexin-A1
BSA: Bovine serum albumin
CCA: Common carotid artery
Co-IP: Coimmunoprecipitation
CNS: Central nervous system
ECA: External carotid artery
FBS: Foetal bovine serum
ICA: Internal carotid artery
LDH: Lactate dehydrogenase
MCAO: Middle cerebral artery occlusion
mNSS: Modified neurological severity score
MWM: Morris water maze
OGD/R: Oxygen–glucose deprivation and reperfusion
RIPA: Radioimmune-precipitation assay
RT-qPCR: Quantitative real-time PCR
SENP6: Sentrin/SUMO-specific protease 6
shRNAs: Short hairpin RNAs
TUNEL: TdT-mediated dUTP-X nick end labelling
TTC: 2,3,5-Triphenyltetrazolium chloride
