# Supplementary figures and images for: SENP6 induces microglial polarization and neuroinflammation through de-SUMOylation of Annexin-A1 after cerebral ischaemia–reperfusion injury
Source: Cell Biosci. 2022 Jul 22;12:113. doi: 10.1186/s13578-022-00850-2 (PMC9308285; doi:10.1186/s13578-022-00850-2)

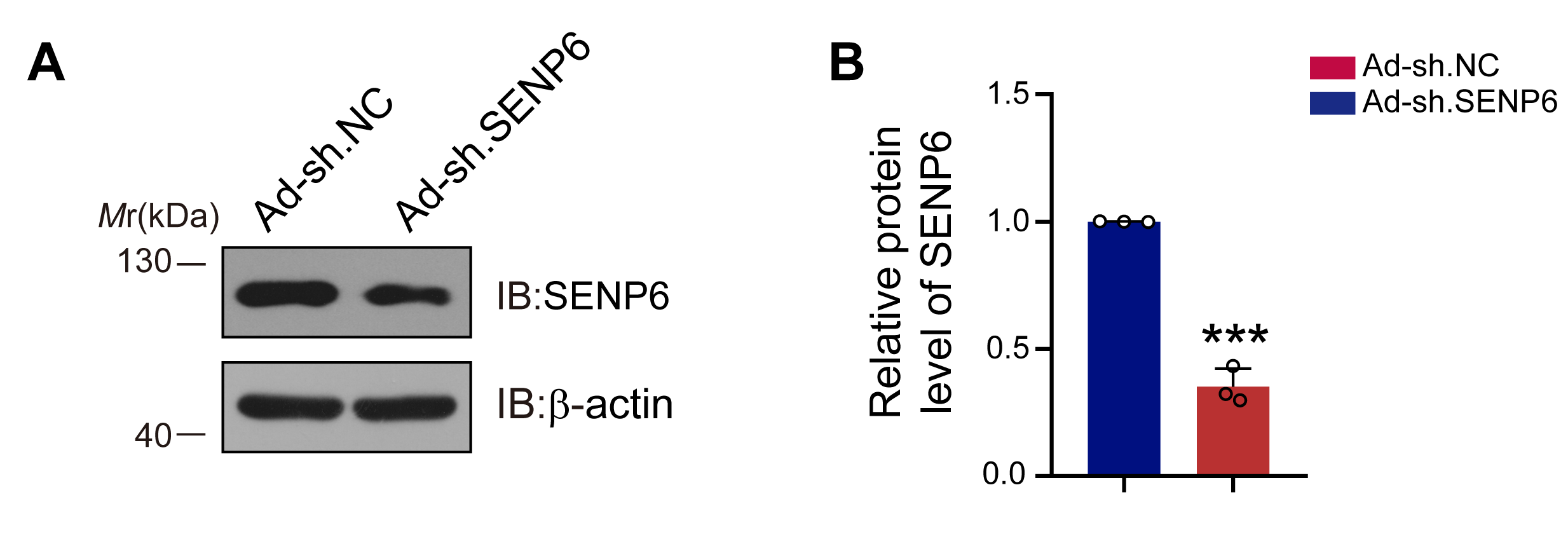

Supplement: Supplementary file 1 — Additional file 1: Fig. S1. The interference efficiency of Ad-sh. SENP6 against mouse SENP6. A Primary cultured microglia were transfected with adenoviral particles expressing either negative control (NC) or SENP6-targeting shRNA for 48 h. Western blotting was performed to examine the blocking efficiency. (B) Statistical analysis of the data shown in Figure S1A. The data are expressed as the means ± S.E.M. from three independent experiments. ***P < 0.001 versus sh.NC. [file 13578_2022_850_MOESM1_ESM.tif]

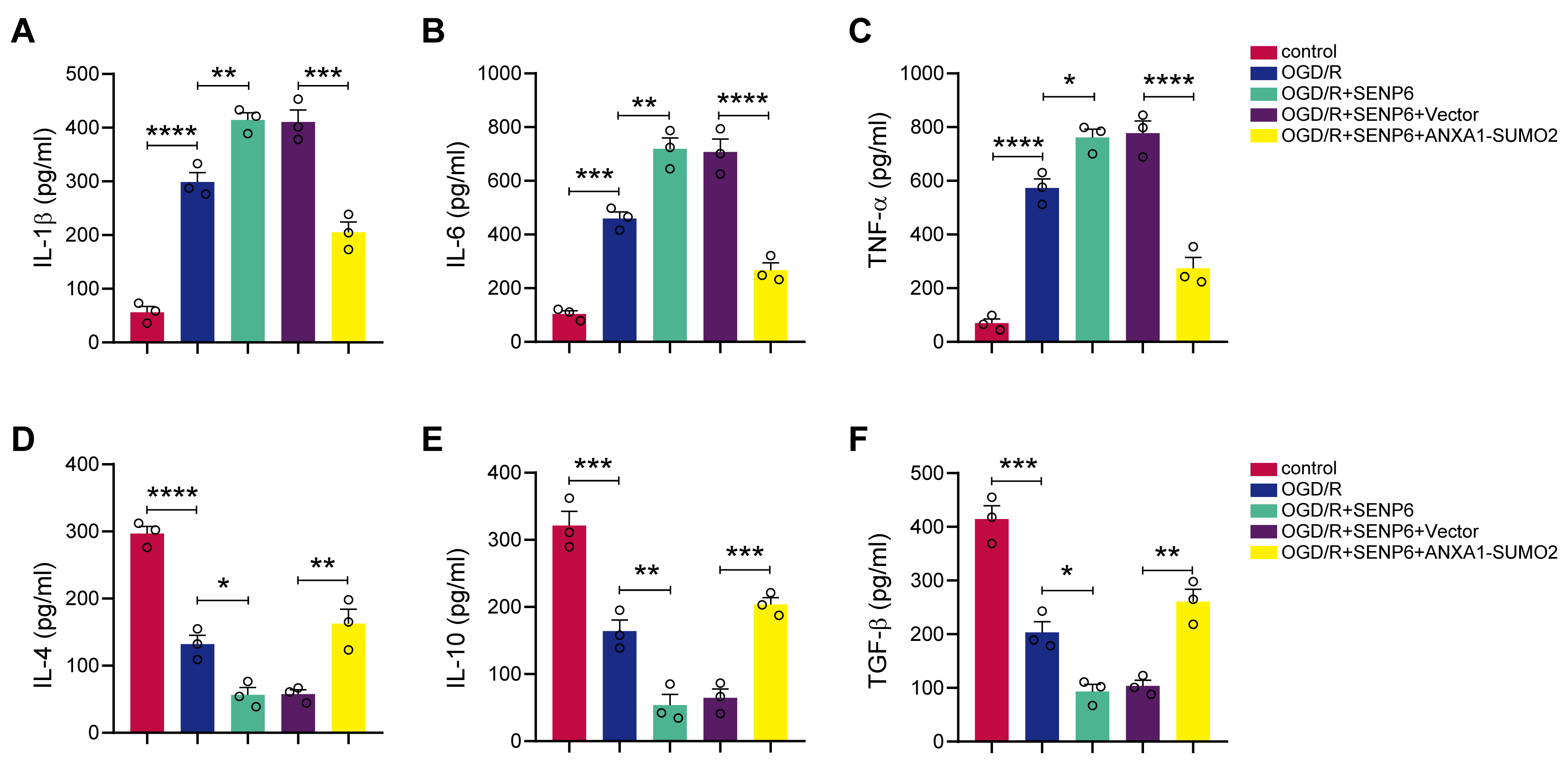

Supplement: Supplementary file 2 — Additional file 2: Fig. S2. SENP6 induced neuroinflammation after ischaemic brain injury depending on its de-SUMOylation of ANXA1. Primary cultured microglia were infected with adenovirus expressing SENP6 or together with ANXA1-SUMO2 following OGD/R treatment. The cytokine levels of OGD/R-induced pro-inflammatory (A to C) and anti-inflammatory (D to F) phenotype marker genes were detected by ELISA assay. Data are presented as the mean ± S.E.M. from three dependent experiments and analysed by one-way ANOVA followed by Tukey’s post hoc test. *P < 0.05, **P < 0.01, ***P < 0.001, and ****P < 0.0001. [file 13578_2022_850_MOESM2_ESM.tif]

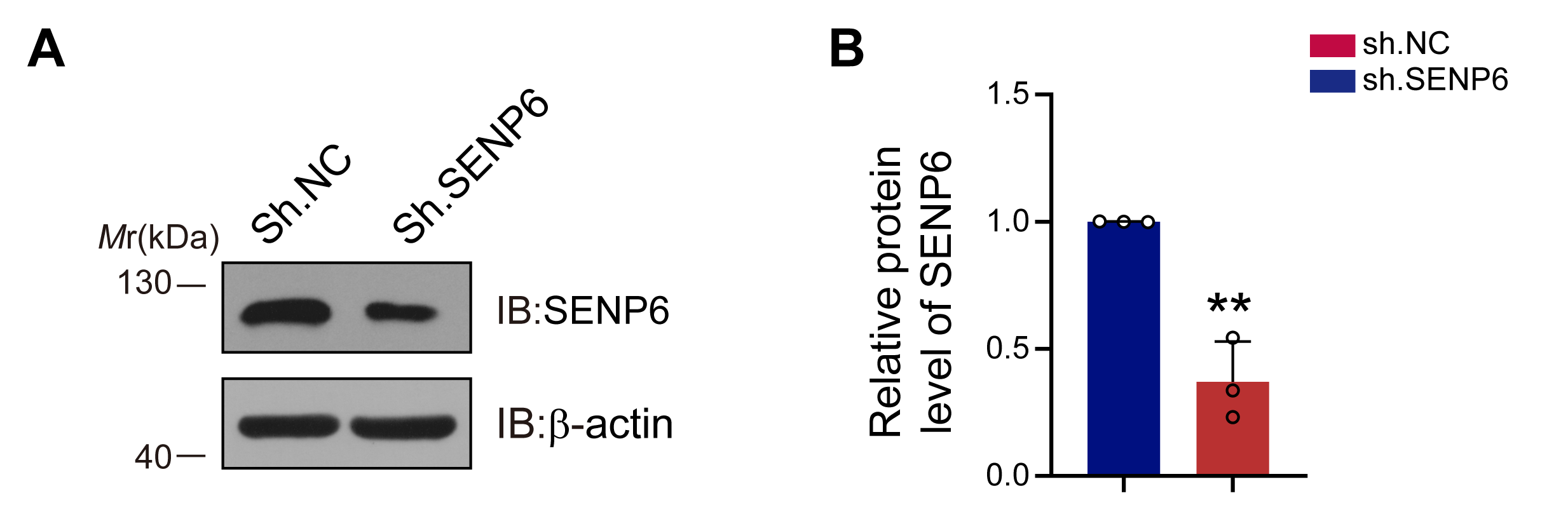

Supplement: Supplementary file 3 — Additional file 3: Fig. S3. The interference efficiency of sh. SENP6 against human SENP6. A HEK293 cells were transfected with shRNA plasmids expressing negative control (NC) or SENP6-targeting shRNA. The knockdown of endogenous SENP6 expression was confirmed by western blot analysis. (B) Statistical analysis of the data shown in Figure S2A. The data are expressed as the means ± S.E.M. from three independent experiments. **P < 0.01 versus sh.NC. [file 13578_2022_850_MOESM3_ESM.tif]

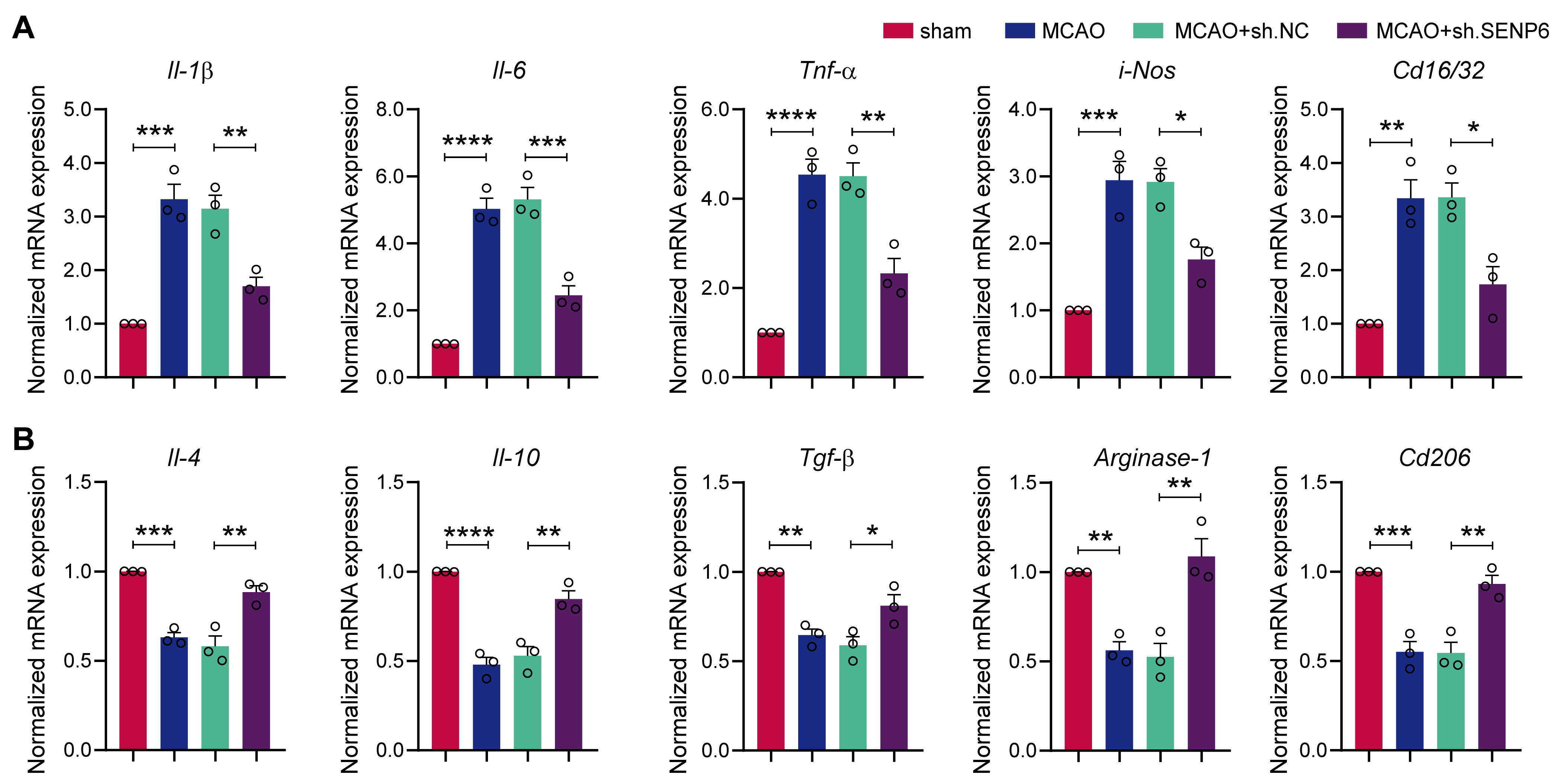

Supplement: Supplementary file 4 — Additional file 4: Fig. S4. Microglia SENP6 silencing promotes an anti-inflammatory phenotype of microglia after cerebral ischaemic injury. (A, B) AAV mediated SENP6 silencing attenuated MCAO-induced mRNA expression of pro-inflammatory marker genes (A) and promoted the mRNA expression of anti-inflammatory marker genes (B) in microglia cells isolated from ischaemic stroke mice. The mRNA levels of pro-inflammatory and anti-inflammatory mediators were detected by RT-qPCR. The results were analysed by one-way ANOVA followed by Tukey’s post hoc test. Data are presented as the mean ± S.E.M., *P < 0.05, **P < 0.01, ***P < 0.001 and ****P < 0.0001. [file 13578_2022_850_MOESM4_ESM.tif]
