# Supplementary material for: SENP6 induces microglial polarization and neuroinflammation through de-SUMOylation of Annexin-A1 after cerebral ischaemia–reperfusion injury
Source: Cell Biosci. 2022 Jul 22;12:113. doi: 10.1186/s13578-022-00850-2 (PMC9308285; doi:10.1186/s13578-022-00850-2)
Supplement: Supplementary file 5 — Additional file 5: Table S1. Primers used in this study. [file 13578_2022_850_MOESM5_ESM.docx]

**Supplementary Table 1. Primers used in this study.**

| Primer name | Primer sequences (5’- 3’) | |
| --- | --- | --- |
|  | Forward | Reverse |
| Quantitative RT-PCR primers | | |
| *Senp6* | ATGCAGACAAAGATGGGGCA | CAGTCTTGCTCCGCCTTACA |
| *Il-1β* | GAAAGACGGCACACCCAC | TGTGACCCTGAGCGACCT |
| *Il-6* | TCTCTGGGAAATCGTGGAA | GATGGTCTTGGTCCTTAGCC |
| *Tnf-α* | ACGGCATGGATCTCAAAGAC | AGATAGCAAATCGGCTGACG |
| *Cxcl1* | GAGCTTGAAGGTGTTGCCCT | CGCGACCATTCTTGAGTGTG |
| *Ccl2* | GCAGGTCCCTGTCATGCTTC | GTGGGGCGTTAACTGCATCT |
| *β-actin* | TTCGTTGCCGGTCCACACCC | GCTTTGCACATGCCGGAGCC |
| Genotyping primers | | |
| *Cx3cr1* Cre | CAACGAGTGATGAGGTTCGCAAG | ACACCAGAGACGGAAATCCATCG |
